# Factors associated with immunological non-response after ART initiation: a retrospective observational cohort study

**DOI:** 10.1186/s12879-024-09021-9

**Published:** 2024-01-29

**Authors:** Heping Zhao, Anping Feng, Dan Luo, Tanwei Yuan, Yi-Fan Lin, Xuemei Ling, Huolin Zhong, Junbin Li, Linghua Li, Huachun Zou

**Affiliations:** 1 Infectious Disease Center, Guangzhou Eighth People’s Hospital, Guangzhou Medical University, Guangzhou, China; 2 School of Public Health (Shenzhen), Sun Yat-sen University, Shenzhen, China; 3 School of Public Health, Fudan University, Shanghai, China; 4 School of Public Health, Southwest Medical University, Luzhou, China; 5 Kirby Institute, University of New South Wales, Sydney, Australia

**Keywords:** PLHIV, INRs, ICRs, IRs, ART

## Abstract

**Background:**

Among people living with HIV (PLHIV) on antiretroviral therapy (ART), the mortality of immunological non-responders (INRs) is higher than that of immunological responders (IRs). However, factors associated with immunological non-response following ART are not well documented.

**Methods:**

We obtained data for HIV patients from the National Free Antiretroviral Treatment Program database in China. Patients were grouped into IRs (CD4 cell count ≥ 350 cells/μl after 24 months’ treatment), immunological incomplete responders (ICRs) (200–350 cells/μl) and INRs (< 200 cells/μl). Multivariable logistic regression was used to assess factors associated with immunological non-response.

**Results:**

A total of 3900 PLHIV were included, among whom 2309 (59.2%) were IRs, 1206 (30.9%) ICRs and 385 (9.9%) INRs. In multivariable analysis, immunological non-response was associated with being male (2.07, 1.39–3.09), older age [40–49 years (vs. 18–29 years): 2.05, 1.29–3.25; 50–59 years: 4.04, 2.33-7.00; ≥ 60 years: 5.51, 2.84–10.67], HBV co-infection (1.63, 1.14–2.34), HCV co-infection (2.01, 1.01–4.02), lower CD4 + T cell count [50–200 cells/μl (vs. 200–350 cells/μl): 40.20, 16.83–96.01; < 50 cells/μl: 215.67, 85.62-543.26] and lower CD4/CD8 ratio (2.93, 1.98–4.34) at baseline. Compared with patients treated with non-nucleoside reverse transcriptase inhibitors (NNRTIs) based regimens, those receiving protease inhibitors (PIs) based regimens were less likely to be INRs (0.47, 0.26–0.82).

**Conclusions:**

We found a sizable immunological non-response rate among HIV-infected patients. Being male, older age, coinfection with HBV and HCV, lower CD4 + T cell count and lower CD4/CD8 ratio are risk factors of immunological non-response, whereas PIs-based regimens is a protective factor.

## Introduction

HIV/AIDS imposes considerable morbidity and mortality worldwide. According to the data of Joint United Nations Programme on HIV and AIDS (UNAIDS), approximately 1.5 million people were newly infected with HIV globally in 2020, and 680,000 people died of HIV/AIDS [1]. Among people living with HIV (PLHIV) the progressive depletion of CD4 + T cells results in immunodeficiency, opportunistic infections, cancer, and death [2]. Antiretroviral therapy (ART) can effectively suppress the HIV viral load and increase the CD4 + T cell count, thus greatly improving the prognosis of PLHIV and prolonging their life expectancy [3–6]. However, a considerable proportion of PLHIV fail to improve their CD4 + T cell level despite of successful viral suppression [7–9], who have an increased risk of developing AIDS-related and non-AIDS-related events, and death [10–12].

The mechanisms for immunological non-response are very complicated and have not been elucidated. The impaired function of bone marrow or thymus has been clearly demonstrated to be associated with immunological non-response [13]. T cells are derived from bone marrow hematopoietic progenitor cells and populate the thymus for differentiation and maturity [14]. The bone marrow abnormalities and the consequent reduced proliferation of progenitor cells that shown in PLHIV can be improved by ART [15], but this is rarely seen in PLHIV with compromised immunological response [16]. Thymic volume is a significant independent predictor for the increase of CD4 + T cells in PLHIV receiving ART [17, 18]. It has been proved that PLHIV with abundant thymic tissue show a faster improvement of naive T cell count compared with PLHIV with smaller thymic tissue [19, 20].

Understanding factors associated with immunological non-response is critical to the improvement of immunological response among PLHIV. Previous studies based on smaller samples have shown that age, sex, coinfections with hepatitis C virus (HCV) and baseline CD4 + T cell count [21–25] were risk factors for immunological non-response. However, the odds ratio of above mentioned factors from different studies were inconsistent and less convincing because of smaller samples, shorter follow-up period and imprecise definition of immunological non-response. Moreover, literature on immunological non-response among PLHIV in China are scarce. In our study, we enrolled PLHIV with different immune responses from Guangzhou to assess factors associated with immunological non-response.

## Methods

### Study design and participants

We performed a retrospective observational cohort study using data retrieved from the National Free Antiretroviral Treatment Program database. This database, which is maintained by the National Center for AIDS/STD Control and China Center for Disease Control and Prevention (China CDC), includes PLHIV who meet China free ART criteria and received free ART. All of the provinces, municipalities and autonomous regions have access to the database within their jurisdiction.

We included all PLHIV who were treated in Guangzhou Eighth People’s Hospital between January 2010 and December 2017, aged 18 years and older, baseline CD4 + T cell count < 350 cells/ul, and had not previously received ART. We excluded PLHIV who: (1) did not have CD4 + or CD8 + T cell count records at baseline or follow-up; (2) route of HIV infection is unknow; (3) were pregnant; (4) had follow-up < 24 months.

This study was approved by the institutional review board of the Guangzhou Eighth People’s Hospital (202033166).

### Procedures

We analyzed the baseline and follow-up information from the National Free Antiretroviral Treatment Program database [26]. Baseline information measured at ART initiation included demographics [sex, age, body-mass index (BMI) and marital status], self-reported route of HIV infection, clinical and laboratory characteristics, date of HIV diagnosis, date of ART start and initial ART regimens.

The immunological non-responders (INRs) were defined as a CD4 + T cell count < 200  cells/ul and HIV viral load < 50 copies/ml after 24 months of ART [27]. Immunological incomplete responders (ICRs) were defined as a CD4 + T cell count within 200~350 cells/ul and HIV viral load < 50 copies/ml after 24 months of ART. Immunological responders (IRs) were defined as a CD4 + T cell count ≥ 350 cells/ul and HIV viral load < 50 copies/ml after 24 months of ART. Comorbidities include one of the following symptoms at baseline: skin lesion, thrush, hairy leukoplakia, persistent diarrhoea, and persistent or intermittent fever. Coinfections include one of the following viruses or bacteria at baseline: tuberculosis, esophageal candidiasis, non-pulmonary cryptococcal, pneumocystis jiroveci pneumonia, disseminated mycosis, cytomegalovirus, extrapulmonary tuberculosis, recurrent severe bacterial pneumonia, zoster and other infections or tumor.

### Statistical analysis

Baseline characteristics were compared using Chi-squared test or Fisher exact test for categorical variables. Multivariable logistic regression analysis was conducted to explore factors associated with immunological non-response. IRs was the reference group. All variables with *P* value less than 0.1 (*P* ≤ 0.1) in the Chi-squared test or proved to be associated with immunological non-response in previous studies were included in the multivariable analysis. Statistical significance was set as a *P* ≤ 0.05 for all analyses. Statistical analyses were performed using R version 4.0.3.

## Results

### Participants selection

**Fig. 1:**
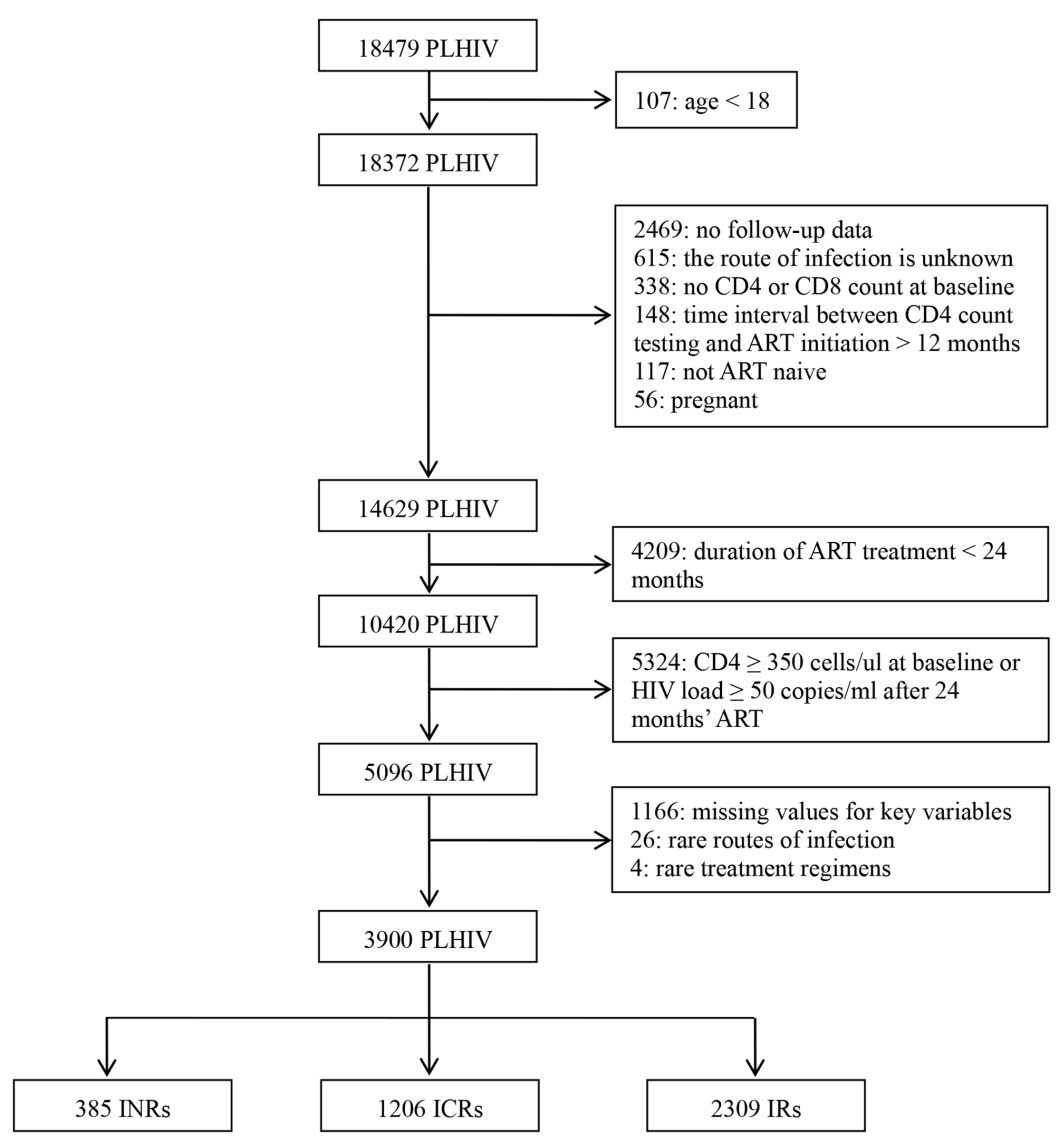
Study flow chart PLHIV, people living with HIV; ART, antiretroviral therapy INRs (Immunological non-responders) were defined as a CD4 + T cell count < 200 cells/ul and HIV viral load < 50 copies/ml after 24 months’ ART ICRs (immunological incomplete responders) were defined as a CD4 + T cell count within 200~350 cells/ul and HIV viral load < 50 copies/ml after 24 months’ ART IRs (immunological responders) were defined as a CD4 + T cell count ≥ 350 cells/ul and HIV viral load < 50 copies/ml after 24 months’ ART

We identified 14629 PLHIV whose CD4 + T cell or CD8 + T cell records were complete at baseline, of whom 4209 were treated with ART less than 24 months, 5324 had a baseline CD4 + T cell count ≥ 350 cells/ul or HIV viral load ≥ 50 copies/ml after 24 months. Of 5096 PLHIV, 1166 missing values for key variables, 4 treated with rare regimens, 26 routes of infection were rare, leaving 3900 PLHIV eligible for analyses, including 2309 (59.2%) IRs, 1206 (30.9%) ICRs and 385 (9.9%) INRs (Fig. 1).

### Patient characteristics at baseline

Patient baseline characteristics are summarized in Table 1. Among all included participants, 3239 (83.1%) were men and 661 (16.9%) were women. 481 PLHIV (12.3%) were older than 50 years old. 720 PLHIV (18.5%) had BMI < 18.5, and 628 PLHIV (16.1%) had BMI ≥ 24.0. 1881 PLHIV (48.2%) were married. 3770 (96.6%) reported acquiring HIV through sexual exposure (1807 heterosexual transmission [46.3%] and 1963 homosexual transmission [50.3%]), and 130 (3.3%) through intravenous drug use.

**Table 1:** Baseline characteristics of PLHIV stratified by CD4 + T cell count after 24 months’ ART (*N* = 3900)

Variable	Overall (%)	INRs (%)	ICRs (%)	IRs (%)	*P* value
	3900(100)	385(9.9)	1206(30.9)	2309(59.2)	
**Sex**					
Male	3239 (83.1)	332 (86.2)	996 (82.6)	1911 (82.8)	0.213
Female	661 (16.9)	53 (13.8)	210 (17.4)	398 (17.2)	
**Age (years)**					
Median (IQR)	34 [27–43]	42 [32–50]	35 [28–44]	32 [26–40]	**< 0.001**
18–29	1329 (34.1)	68 (17.7)	365 (30.3)	896 (38.8)	**< 0.001**
30–39	1273 (32.6)	101 (26.2)	382 (31.7)	790 (34.2)	
40–49	817 (20.9)	115 (29.9)	293 (24.3)	409 (17.7)	
50–59	324 (8.3)	64 (16.6)	111 (9.2)	149 (6.5)	
≥ 60	157 (4.0)	37 (9.6)	55 (4.6)	65 (2.8)	
**BMI**					
Median (IQR)	20.8 [19.0-22.9]	19.9 [18.3–22.1]	20.4 [18.7–22.4]	21.1 [19.4–23.2]	**< 0.001**
< 18.5	720 (18.5)	105 (27.3)	266 (22.1)	349 (15.1)	**< 0.001**
18.5–24.0	2552 (65.4)	243 (63.1)	781 (64.8)	1528 (66.2)	
≥ 24.0	628 (16.1)	37 (9.6)	159 (13.2)	432 (18.7)	
**Marital status**					
Married	1881 (48.2)	258 (67.0)	637 (52.8)	986 (42.7)	**< 0.001**
Unmarried	1828 (46.9)	107 (27.8)	500 (41.5)	1221 (52.9)	
Divorced	191 (4.9)	20 (5.2)	69 (5.7)	102 (4.4)	
**Infection route**					
Heterosexual	1807 (46.3)	234 (60.8)	639 (53.0)	934 (40.5)	**< 0.001**
Homosexual	1963 (50.3)	132 (34.3)	516 (42.8)	1315 (57.0)	
Intravenous drug use	130 (3.3)	19 (4.9)	51 (4.2)	60 (2.6)	
**HBV**					
Positive	495 (12.7)	72 (18.7)	172 (14.3)	251 (10.9)	**< 0.001**
Negative	3405 (87.3)	313 (81.3)	1034 (85.7)	2058 (89.1)	
**HCV**					
Positive	184 (4.7)	29 (7.5)	69 (5.7)	86 (3.7)	**0.001**
Negative	3716 (95.3)	356 (92.5)	1137 (94.3)	2223 (96.3)	
**Baseline CD4 + cell count** (cells/μl)					
Median (IQR)	203 [88–271]	28 [10–73]	122 [38–198]	250 [194–297]	**< 0.001**
< 50	718 (18.4)	245 (63.6)	346 (28.7)	127 (5.5)	**< 0.001**
50–200	1184 (30.4)	134 (34.8)	562 (46.6)	488 (21.1)	
200–350	1998 (51.2)	6 (1.6)	298 (24.7)	1694 (73.4)	
**Baseline CD4+/CD8 ratio**					
Median (IQR)	0.22 [0.12–0.32]	0.06 [0.03–0.13]	0.15 [0.07–0.25]	0.27 [0.18–0.36]	**< 0.001**
< 0.2	1721 (44.1)	341 (88.6)	744 (61.7)	636 (27.5)	**< 0.001**
≥ 0.2	2179 (55.9)	44 (11.4)	462 (38.3)	1673 (72.5)	
**HIV load** (copies/ml)					
< 10,000	88 (13.5)	6 (7.9)	23 (10.8)	59 (16.3)	**< 0.001**
10,000–30,000	124 (19.1)	8 (10.5)	33 (15.6)	83 (22.9)	
30,000–100,000	192 (29.5)	16 (21.1)	63 (29.7)	113 (31.2)	
> 100,000	246 (37.8)	46 (60.5)	93 (43.9)	107 (29.6)	
**Time before ART (months)**					
Median (IQR)	1.0 [0.5–6.4]	0.9 [0.5–2.2]	1.0 [0.5–3.9]	1.1 [0.5–8.8]	**< 0.001**
< 1	1858 (47.6)	203 (52.7)	587 (48.7)	1068 (46.3)	**< 0.001**
1–6	1038 (26.6)	116 (30.1)	358 (29.7)	564 (24.4)	
6–12	250 (6.4)	19 (4.9)	59 (4.9)	172 (7.4)	
≥ 12	754 (19.3)	47 (12.2)	202 (16.7)	505 (21.9)	
**ART regimens**					
NRTIs + NNRTIs	3465 (88.8)	313 (81.3)	1073 (89.0)	2079 (90.0)	**< 0.001**
NRTIs + PIs	260 (6.7)	22 (5.7)	74 (6.1)	164 (7.1)	
NRTIs + INSTIs	78 (2.0)	18 (4.7)	29 (2.4)	31 (1.3)	
NRTIs	97 (2.5)	32 (8.3)	30 (2.5)	35 (1.5)	
**SMZ-TMP**					
Yes	1515 (38.8)	312 (81.0)	705 (58.5)	498 (21.6)	**< 0.001**
No	2385 (61.2)	73 (19.0)	501 (41.5)	1811 (78.4)	
**Comorbidities**					
Yes	495 (12.7)	117 (30.4)	205 (17.0)	173 (7.5)	**< 0.001**
No	3405 (87.3)	268 (69.6)	1001 (83.0)	2136 (92.5)	
**Coinfections**					
Yes	380 (9.7)	80 (20.8)	165 (13.7)	135 (5.8)	**< 0.001**
No	3520 (90.3)	305 (79.2)	1041 (86.3)	2174 (94.2)	

PLHIV, people living with HIV; ART, antiretroviral therapy; BMI, body mass index

HBV, hepatitis B virus. HCV, hepatitis C virus

INRs (Immunological non-responders) were defined as a CD4 + T cell count < 200 cells/ul and HIV viral load < 50 copies/ml after 24 months’ ART

ICRs (immunological incomplete responders) were defined as a CD4 + T cell count within 200~350 cells/ul and HIV viral load < 50 copies/ml after 24 months’ ART

IRs (immunological responders) were defined as a CD4 + T cell count ≥ 350 cells/ul and HIV viral load < 50 copies/ml after 24 months’ ART

Time before ART, time interval between HIV diagnosis and ART initiation

NRTIs, nucleoside reverse transcriptase inhibitors; NNRTIs, non-nucleoside reverse transcriptase inhibitors; PIs, protease inhibitors; INSTIs, integrase strand transfer inhibitors; SMZ-TMP, sulfamethoxazole and trimethoprim

Comorbidities include one of the following symptoms at baseline: skin lesion, thrush, hairy leukoplakia, persistent diarrhoea, and persistent or intermittent fever

Coinfections include one of the following viruses or bacteria at baseline: tuberculosis, esophageal candidiasis, non-pulmonary cryptococcal, pneumocystis jiroveci pneumonia, disseminated mycosis, cytomegalovirus, extrapulmonary tuberculosis, recurrent severe bacterial pneumonia, zoster and other infections or tumor

IQR = interquartile range

Compared with IRs, more older people were ICRs and INRs (*P* < 0.001). The percentage of overweight PLHIV (BMI ≥ 24.0) in the IRs group (18.7%) is the highest among the three groups. A total of 1315 PLHIV (57.0%) in the IRs group reported acquiring HIV through homosexual transmission, significantly higher than that in the ICRs and INRs group. The co-infection rates of hepatitis B virus (HBV) (18.7%) and HCV (7.5%) in the INRs group were significantly higher than that in the IRs group. Similarly, the percentage of previous using sulfamethoxazole and trimethoprim (SMZ-TMP) (81.0%) in the INRs group was significantly higher than that in the IRs (21.6%) group. The baseline CD4 + T cell count and baseline CD4/CD8 ratio in the INRs group were significantly lower than those in the IRs group. The percentage of HIV load more than 100,000 in the INRs group (60.5%) was significantly higher than that in the IRs group (29.6%). Meanwhile, the rates of comorbidities (30.4%) and coinfections (20.8%) in INRs were significantly higher than that in the IRs group (7.5% and 5.8%). As a result, sex, age, BMI, marital status, infection route, HBV, HCV, CD4, CD4/CD8, time before ART, ART regimens, SMZ-TMP, comorbidities and coinfections were included in the multivariable analysis.

### Factors associated with immunological non-response

Table 2 shows the factors associated with immunological incomplete response and immunological non-response at baseline. In the multivariable logistic regression analysis, immunological non-response was associated with being male (adjusted odds ratio [aOR], 2.07; 95% confidence interval [CI], 1.39–3.09), older age (40–49 years vs. 18–29 years: 2.05, 1.29–3.25; 50–59 years vs. 18–29 years: 4.04, 2.33-7.00; ≥ 60 years vs. 18–29 years: 5.51, 2.84–10.67), HBV co-infection (1.63, 1.14–2.34), HCV co-infection (2.01, 1.01–4.02), lower baseline CD4 + T cell count (50–200 cells/μl vs. 200–350 cells/μl: 40.20, 16.83–96.01; < 50 cells/μl vs. 200–350 cells/μl: 215.67, 85.62-543.26) and lower baseline CD4/CD8 ratio (< 0.2 vs. ≥ 0.2: 2.93, 1.98–4.34). Compared with patients treated with non-nucleoside reverse transcriptase inhibitors (NNRTIs) based regimens, those receiving protease inhibitors (PIs) based regimens were less likely to be INRs (nucleoside reverse transcriptase inhibitors [NRTIs] + PIs vs. NRTIs + NNRTIs: 0.47, 0.26–0.82).

**Table 2:** Factors associated with immunological incomplete response and immunological non-response among PLHIV

Variable	ICRs			INRs		
	aOR	95% CI	*P* value	aOR	95% CI	*P* value
**Sex**						
Female	1.00			1.00		
Male	1.50	1.17–1.92	**0.001**	2.07	1.39–3.09	**< 0.001**
**Age (years)**						
18–29	1.00			1.00		
30–39	0.91	0.74–1.13	0.406	1.06	0.71–1.58	0.789
40–49	1.16	0.88–1.52	0.290	2.05	1.29–3.25	**0.002**
50–59	1.29	0.90–1.85	0.162	4.04	2.33-7.00	**< 0.001**
≥ 60	1.41	0.88–2.26	0.152	5.51	2.84–10.67	**< 0.001**
**BMI**						
< 18.5	1.15	0.93–1.43	0.209	1.26	0.91–1.75	0.163
18.5–24.0	1.00			1.00		
≥ 24.0	0.78	0.62–0.98	**0.035**	0.68	0.44–1.04	0.074
**Marital status**						
Married	1.00			1.00		
Unmarried	0.97	0.77–1.22	0.797	0.82	0.55–1.21	0.310
Divorced	1.15	0.79–1.68	0.461	0.84	0.46–1.55	0.584
**Infection route**						
Heterosexual	1.00			1.00		
Homosexual	0.84	0.68–1.05	0.131	1.34	0.93–1.91	0.113
Intravenous drug use	0.78	0.44–1.39	0.407	0.51	0.23–1.17	0.115
**HBV**						
Positive	1.24	0.98–1.58	0.079	1.63	1.14–2.34	**0.008**
Negative	1.00			1.00		
**HCV**						
Positive	1.27	0.79–2.06	0.328	2.01	1.01–4.02	**0.047**
Negative	1.00			1.00		
**Baseline CD4 + cell count** (cells/μl)						
200–350	1.00			1.00		
50–200	5.14	4.05–6.52	**< 0.001**	40.20	16.83–96.01	**< 0.001**
< 50	10.38	7.32–14.70	**< 0.001**	215.67	85.62-543.26	**< 0.001**
**Baseline CD4+/CD8 ratio**						
≥ 0.2	1.00			1.00		
< 0.2	1.57	1.29–1.89	**< 0.001**	2.93	1.98–4.34	**< 0.001**
**Time before ART (months)**						
< 1	1.00			1.00		
1–6	1.15	0.95–1.40	0.158	1.05	0.77–1.43	0.771
6–12	0.89	0.62–1.26	0.504	1.27	0.69–2.34	0.447
≥ 12	0.82	0.66–1.02	0.076	0.75	0.50–1.11	0.151
**ART regimens**						
NRTIs + NNRTIs	1.00			1.00		
NRTIs + PIs	0.70	0.49–0.98	**0.037**	0.47	0.26–0.82	**0.008**
NRTIs + INSTIs	0.71	0.40–1.29	0.265	0.84	0.41–1.73	0.632
NRTIs	0.62	0.35–1.09	0.098	1.13	0.61–2.10	0.698
**SMZ-TMP**						
Yes	1.00			1.00		
No	0.91	0.72–1.14	0.407	0.79	0.56–1.11	0.176
**Comorbidities**						
Yes	1.00			1.00		
No	1.24	0.93–1.65	0.145	1.19	0.83–1.72	0.348
**Coinfections**						
Yes	1.00			1.00		
No	0.97	0.72–1.31	0.851	1.14	0.77–1.69	0.528

PLHIV, people living with HIV; ART, antiretroviral therapy; BMI, body mass index

HBV, hepatitis B virus. HCV, hepatitis C virus

ICRs (immunological incomplete responders) were defined as a CD4 + T cell count within 200~350 cells/ul and HIV viral load < 50 copies/ml after 24 months’ ART

INRs (Immunological non-responders) were defined as a CD4 + T cell count < 200 cells/ul and HIV viral load < 50 copies/ml after 24 months’ ART

Time before ART, time interval between HIV diagnosis and ART initiation

NRTIs, nucleoside reverse transcriptase inhibitors; NNRTIs, non-nucleoside reverse transcriptase inhibitors; PIs, protease inhibitors; INSTIs, integrase strand transfer inhibitors; SMZ-TMP, sulfamethoxazole and trimethoprim

Comorbidities include one of the following symptoms at baseline: skin lesion, thrush, hairy leukoplakia, persistent diarrhoea, and persistent or intermittent fever

Coinfections include one of the following viruses or bacteria at baseline: tuberculosis, esophageal candidiasis, non-pulmonary cryptococcal, pneumocystis jiroveci pneumonia, disseminated mycosis, cytomegalovirus, extrapulmonary tuberculosis, recurrent severe bacterial pneumonia, zoster and other infections or tumor

## Discussion

In this analysis from a large observational cohort, we found that being male, older age, HBV co-infection, HCV co-infection, lower CD4 + T cell and lower CD4/CD8 ratio at baseline were associated with immunological non-response. Compared with patients treated with NNRTIs-based regimens, those receiving PIs-based regimens were less likely to be INRs.

Compared to younger PLHIV, older PLHIV have poorer immune recovery. After the introduction of ART, several studies reported that immunological response was less marked in older PLHIV [28, 29]. Similarly, a study performed in the EuroSIDA cohort in which older age was associated with a lower increase in the CD4 + T cell count after 12 months of ART [30]. This could be explained because older PLHIV who initiate ART with an advanced degree of clinical progression and their CD4 + T cell levels reach an earlier plateau than that in younger PLHIV after ART. Besides, the effect of age on immune recovery may be related to the poorer thymic function and lower ART adherence in older PLHIV [31]. However, a prospective case-control study of immunological recovery in 596 PLHIV demonstrated that although older PLHIV (≥ 50) present a more severe HIV infection, they can achieve the same immunological success as their younger counterparts (20–35) [32]. These differences may come from sample size, age stratification and observation duration, for the PLHIV in the prospective case-control study mentioned above were followed up for 6 years, which was significantly longer than our 2-year follow-up period. Of note, we found that time before ART was not proven to be a risk factor for CD4 restoration, which probably because that all the included patients were late presenters, so it is not so relevant when treatment was started because they have probably acquired HIV more than 6–8 years before HIV was diagnosed.

We found that HBV and HCV co-infection significantly increase the risk of immunological non-response. HIV shares similar routes of transmission with HBV and HCV, co-infection with HBV and HCV are very common in PLHIV. HCV or HBV co-infection could affect the timing of ART initiation and broaden the indication for anti-HBV or anti-HCV treatment. The issue has been investigated in several previous studies with conflicting results. A swiss HIV cohort study found that HCV seropositivity was associated with a smaller CD4 + T cell recovery [24], which is consistent with findings from our study. However, Peters et al. found that HCV co-infection had no significant effect on CD4 + T cell number recovery as long as viral load was suppressed to the maximum extent [33]. It is probably because that Peters et al. assessed the influence of HCV genotype on the CD4 + T cell recovery, and differences in adherence between HCV-infected and HCV-uninfected PLHIV may contribute to the disparate findings. In our study, we did not identify HCV genotype and assess the role of ART adherence on immunological response. Of note, we found HBV co-infection was the risk factor that rarely reported in previous studies, which serves as a reminder for physicians to pay more attention to PLHIV coinfected with HBV.

In our cohort, we found that baseline CD4 + T cell count < 50 cells/μl greatly increase the risk of immunological non-response, indicating that recommend PLHIV initiate ART earlier could lead to an increase in the number who achieve immunological response, since a low CD4 count at baseline is mainly the result of late diagnosis and treatment. Likewise, a study of immunological recovery in 861 PLHIV demonstrated that most PLHIV with CD4 + T cell count < 200 cells/μl before ART failed to achieve a CD4 + T cell count of > 500/ul [34]. PLHIV with low CD4 + T cell count at diagnosis were supposed to be late presenters, previous study showed that late presenters had a higher level of T cell activation than those with higher baseline CD4 + T cell count [35], which may contribute to the failure restoration of CD4 + T cell. we found the lower baseline CD4/CD8 ratio was associated with a higher risk of INRs, which is in accordance with a previous report [36]. This finding suggests that imbalance between CD4 and CD8 + T cell populations at baseline may be an important predictor to identify PLHIV with the worse prognosis. Of note, our data found that PIs-containing regimens reduce the risk of immunological non-response, which might suggest that PIs-containing regimens is a clinical benefit factor, probably because of a less toxic effect of PIs. However, previous study showed no differences in terms of immunological response between PIs and NNRTIs [30], which probably because the baseline CD4 + T cell count were significantly different between different ART regimen groups in previous study, while our participants were naïve to ART and had < 350 CD4 + T cell count at ART initiation. We did not see better immune recovery in PLHIV with INSTIs when compared to other ART regimens, which was probably due to the low proportion of PLHIV who started with INSTIs in this cohort.

Our analysis had some limitations. Firstly, we restricted INRs to be within 2 years, which may lead to missed INRs if longer follow-up period were given. However, most PLHIV whose CD4 + T cell count should have already reached their maximum potential within 2 years [27]. For example, Richard conducted a longitudinal observational study for six years to characterize the increase of CD4 + T cell count in PLHIV in clinical practice who maintained sustained viral suppression, and he found that CD4 + T cell increased most rapidly in the first two years, after which the rate of increase was slow and gradually reached a plateau [28]. Secondly, we found that the number of CD4 + T cell at baseline had an effect on INRs, but we were unable to differentiate CD4 + T cell subsets. Different CD4 + T cell subgroups have different roles. For example, Th17 cells are T cells with proinflammatory properties, and they may play an important role in immunological non-response [37].

In conclusion, our results based on a large cohort show that being male, older age, HBV/HCV co-infection, lower baseline CD4 + T cell count and lower CD4/CD8 ratio increase the risk of immunological non-response. Receiving PIs-containing regimens tend to be protective factor.

## Data Availability

The datasets generated and/or analysed during the current study are not publicly available, but are available from the corresponding author on reasonable request.
